# KRAS Status is Associated with Metabolic Parameters in Metastatic Colorectal Cancer According to Primary Tumour Location

**DOI:** 10.1007/s12253-020-00850-y

**Published:** 2020-06-27

**Authors:** M. Tabuso, M. Christian, P. K. Kimani, K. Gopalakrishnan, R. P. Arasaradnam

**Affiliations:** 1 Department of Gastroenterology, University Hospital Coventry and Warwickshire, Clifford Bridge Road, Coventry, CV2 2DX UK; 2 Warwick Medical School, University of Warwick, Coventry, CV4 7AL UK; 3 School of Science and Technology, Nottingham Trent University, Nottingham, NG11 8NS UK; 4 Department of Pathology, University Hospitals of Coventry and Warwickshire NHS Trust, Clifford Bridge Road, Coventry, CV2 2DX UK; 5 The University of Warwick, School of life Sciences, Coventry, CV4 7AL UK; 6 Faculty of Health and Life Sciences, University of Coventry, Priory Street, Coventry, CV1 5BF UK; 7 University of Leicester, Leicester, LE1 7RH UK

**Keywords:** Colorectal cancer, KRAS, Metabolic syndrome, Tumour location, Lipids

## Abstract

Colorectal cancer (CRC) is characterized by complex interplay between macroenvironmental factors and tumour microenvironment, leading to variable outcomes in CRC patients. To date, there is still a need to identify macroenvironment/microenvironment factors that could define subgroup of patients that would benefit from specific anti-cancer treatment in order to improve patient selection for individualized targeted-based therapy. Aim of this study was to evaluate associations between metabolic parameters and KRAS status in metastatic CRC (mCRC) according to a new tumour site classification. Retrospective data were extracted from a total of 201 patients diagnosed with mCRC between 2012 and 2017 extracted from an established CRC database at our tertiary institute. Clinical-pathological data, including age, gender, BMI, hypertension, diabetes, pre-CRC diagnosis serum lipid levels and KRAS status were recorded. Categorical characteristics were compared using chi-squared test. Continuous characteristics were compared using Mann-Whitney U test. Log rank test was used to compare hazards for survival. In all comparisons, a two-sided *P* value <0.05 was considered statistically significant. Out of 201 patients, 170 patients with complete serum lipid profile were included in the analysis. In recto-sigmoid cancers there was a statistically significant association between high cholesterol:high-density lipoprotein (chol:HDL) ratio and KRAS mutation (OR 2.69, 95% CI 1.1–6.4, *p* = 0,02). In non recto-sigmoid cancers, high cholesterol was associated with KRAS WT (OR 0.39, CI 0.15–0.97, *p* = 0.04). In 22 patients with KRAS mutated recto-sigmoid cancer stage IV at diagnosis normal chol:HDL ratio was associated with a trend to better survival (*p* = 0.06). High chol:HDL ratio was significantly associated with KRAS mutated metastatic recto-sigmoid cancers. A subgroup of mCRC patients with KRAS mutated recto-sigmoid cancer may benefit from optimal lipid lowering treatment.

**Electronic supplementary material:**

The online version of this article (10.1007/s12253-020-00850-y) contains supplementary material, which is available to authorized users.

## Introduction

Colorectal cancer (CRC) is a heterogeneous disease characterized by complex interplay between genetic and epigenetic modifications within tumour and environmental factors. Biologic modifications also occur in the macroscopically normal colonic mucosa adjacent to the neoplastic lesion, known as field cancerization [1]. Obesity and metabolic syndrome (MetS) are established risk factors of CRC incidence and mortality [2, 3]. Recently, the role of dysfunctional adipose tissue in cancer progression has emerged [4, 5]. Nevertheless, interactions between genetic, epigenetic and environmental factors in CRC risk and carcinogenesis remain poorly characterized.

Despite recent advances in cancer therapies, CRC remains the second cause of cancer death in the Western world [6]. To date, there is still a need to define subgroup of patients that would benefit from specific anti-cancer treatment in order to improve patient selection for individualized targeted-based therapy.

Around 20% of CRC patients present with metastatic disease and approximately 25%–30% of patients with CRC stage II/III develop recurrence within 5 years of curative intent surgery [7]. Current predictive and prognostic systems, consisting in tumour, node, metastasis (TNM) classification combined with molecular biomarkers lack accuracy. The most frequent point mutations in KRAS codon 12 and 13 have been recognized as predictive markers of resistance to anti-epidermal growth factor receptor (EGFR) targeted therapies, cetuximab and panitumumab, as adjuvant treatment in advanced disease in combination with cytotoxic chemotherapy [8]. However, only 40–60% of patients with wild type KRAS tumours respond to anti EGFR therapy. A new classification has been reported for clinical classification and subtype-based targeted therapies based on four consensus molecular subtypes (CSM), considered a more robust classification than the current TNM system [9]. Primary tumour location (right sided versus left sided) has recently emerged as prognostic and predictive factor in patients with KRAS wild type mCRC treated with chemotherapy and EGFR directed antibodies, with worse survival in patients with right sided colon cancer and KRAS wild type [10, 11], whilst KRAS mutations have been associated with worse survival only in left sided cancer [12]. However, current left/right sided colon cancer classification is still debated. Reliable prognostic and predictive biomarkers to guide treatment in patients with stage IV CRC are needed.

Traditional epidemiologic research has focused on risk factors associated with CRC without considering CRC genetic and epigenetic heterogeneity. A new area of research termed “molecular pathologic epidemiology” has detected epigenetic modifications associated with obesity, providing insights into the relationship between obesity and molecular variants in CRC [13]. Associations between environmental factors and KRAS status have not been extensively investigated. Only a few studies have reported inconsistent findings regarding associations between BMI/metabolic parameters and KRAS status [14–18].

In the present study, we evaluated associations between clinical, metabolic and molecular parameters (KRAS status) in metastatic CRC (mCRC) patients according to a novel classification for primary tumour location. There is recent evidence suggesting similar biologic features in sigmoid colon and rectal cancers [19, 20]. We clustered CRCs according to primary location at rectum and sigmoid colon (recto-sigmoid cancers) and at ascending, transverse and proximal descending colon (non recto-sigmoid cancers).

Identifying associations between macro-environmental features and CRC molecular profile is critical to identify adjunctive prognostic and predictive markers of response to therapy and potential new therapeutic targets.

## Methods

Research Governance approval was obtained from Research and Development department at University Hospitals Coventry and Warwickshire.

### Study Population

All mCRC patients diagnosed between January 2012 and July 2017 at University Hospitals Coventry and Warwickshire were retrieved from a CRC database. Patients with histologically confirmed diagnosis of CRC and available KRAS status on histology report were included in this study. Clinical-pathological data including age, gender, BMI, hypertension, diabetes and lipid profile consisting in pre CRC diagnosis median values of total serum cholesterol, triglyceride, high-density lipoprotein cholesterol (HDL-chol), cholesterol:high-density lipoprotein (chol:HDL) ratio, low-density lipoprotein-cholesterol (LDL-chol) were retrospectively collected from clinical records. Patients were considered with raised lipid profile according to the following cut-off values: cholesterol ≥5 mmol/l, chol:HDL ratio ≥ 3.5, LDL-chol ≥1.8 mmol/l, HDL-chol <1.03 mmol/l in men and < 1.29 mmol/l in women, triglycerides ≥1.7 mmol/l. Cut off value for HbA1c was 48 mmol/mol, which is the cut off point for diagnosis of diabetes. Pathological data including CRC anatomic location, stage and KRAS status were also retrieved. KRAS mutation analysis was performed at University Hospitals Birmingham. DNA was extracted from paraffin embedded sections. KRAS mutations in codon 12, 13 and 61 were analyzed employing KRAS Pyro Kit, according to the manufacturer protocol (Quiagen, Germany). KRAS mutations in codon 146 were analyzed employing RAS extension Pyro Kit, according to the manufacturer protocol (Quiagen, Germany).

### CRC Classification

Tumours were classified as non recto-sigmoid cancers if located at ascending colon, transverse colon and proximal descending colon, and recto-sigmoid cancers if located at rectum and sigmoid colon. All CRCs were classified as KRAS mutated or wild type (WT). Cancer stage was classified into II, III and IV according to the TNM staging system of the American Joint Committee on Cancer (AJCC) 7th Edition [21].

### Patient Classification

Patients were classified in relation to body mass index (BMI) in three groups: normal weight patients (BMI ≥18.5 ≤ 24.9), overweight (BMI ≥25 ≤ 29.9), obese patients (BMI ≥30). For Asian patients we employed cut off for obesity of ≥27.5 according to WHO Expert Consultation [22].

Hypertension was defined as use of any antihypertensive medication. Diabetes mellitus was defined as use of any diabetes medication.

Patients were classified in two groups according to presence or absence of MetS. Patients were considered to have MetS when they presented central obesity and two or more criteria according to the International Diabetes Federation criteria (IDF) [23] (Supplementary File Table 1).

### Statistical Analysis

IBM SPSS v 24.0 /2016 was used for statistical analysis. Categorical characteristics were compared using the chi-squared test or Fisher’s exact test, as appropriate. Mann-Whitney U test was used to compare continuous characteristics between KRAS mutated and wild type groups. Means and standard deviations (SD) of continuous characteristics for KRAS mutated and WT groups were reported. The odds ratio (OR) (mutated to WT) comparing the presence of characteristic such as raised cholesterol and 95% confidence intervals (CI) were reported. For the analysis including multiple characteristics, a multivariable logistic regression model was used. Patient survival curves were plotted using the Kaplan-Meier method. Log rank test was used to compare hazards of death between patients with high and low chol:HDL ratio in CRC stage III and stage IV at diagnosis according to primary tumour location. A two-sided *P* value <0.05 was considered statistically significant.

## Results

Out of approximately 1000 CRC patients, 201 patients had histological diagnosis of CRC with KRAS status available on histology report and partial lipid profile (total serum cholesterol, chol:HDL ratio and LDL-cholesterol). Out of 201 patients, 170 had complete lipid profile (total serum cholesterol, triglyceride, HDL-chol, chol:HDL ratio and LDL-cholesterol). Out of 170 patients, 68 (40%) were females and 102 (60%) were males. The median age at diagnosis was 66 years (SD11.30). 28 patients (16.47%) had cancer located at caecum, 23 (13.5%) at ascending colon, 7 (4.1%) at hepatic flexure, 11 (6.5%) at transverse colon, 4 (2.3%) at splenic flexure, 4 (2.3%) at descending colon, 30 (17.6%) at sigmoid colon, 63 (37%) at rectum. 99 patients (58.23%) exhibited KRAS mutations and 71 (41.76%) KRAS WT. 50% of recto-sigmoid cancers exhibited KRAS mutation. Equally, 50% of non recto-sigmoid cancers exhibited KRAS mutation. Demographic characteristics of the cohort are represented in Table 1.

**Table 1:** Demographic characteristics of the cohort of patients with mCRC (*n* = 170)

Characteristic	All patients	KRAS mutation
Age at diagnosis (years), mean ± SD	66.28 ± 11.3	
Gender		
Male, n (%)	102 (60)	
Female, n (%)	68 (40)	
Ethnicity		
Caucasian, n (%)	157 (92.3)	
Non-caucasian, n (%)	13 (18.5)	
BMI (kg/m^2^), mean ± SD	27.7 (6.0)	
Overweight/obese, n (%)	119 (70)	
Metabolic Syndrome according to IDF definition		
Non-sigmoid rectal cancers, n (%)	18 (10.5)	
Sigmoid rectal cancers, n (%)	22 (12.9)	
Metabolic syndrome according to modified IDF definition (central obesity assumed if BMI ≥29)		
Non-sigmoid rectal cancers, n (%)	20 (11.7)	
Sigmoid rectal cancers, n (%)	24 (14.1)	
Diabetes, n (%)		
Dyslipidaemia, n (%)	38 (22.3)	
Dyslipidaemia in treatment with statins, n (%)	81 (47.6)	
Hypertension, n (%)	74 (91.3)	
Cancer sub-location	110 (64.7)	
Caecum, n (%)	28 (16.4)	19 (11.2)
Ascending colon, n (%)	23 (13.)	10 (5.8)
Hepatic flexure, n (%)	7 (4.1)	1 (0.5)
Transverse colon, n (%)	11 (6.5)	2 (1.2)
Splenic flexure, n (%)	4 (2.3)	2 (1.2)
Descending colon, n (%)	4 (2.3)	2 (1.2)
Sigmoid colon, n (%)	30 (17.6)	9 (5.3)
Rectum, n (%)	63 (17)	28 (16.5)
Definition of primary tumour location		
Non-sigmoid rectal cancers, n (%)	78 (45.8)	
Sigmoid rectal cancers, n (%)	92 (54.1)	
Stage at diagnosis		
II, n (%)	14 (8.2)	
III, n (%)	75 (44.1)	
IV, n (%)	81 (47.6)	
KRAS status		
Wild type, n (%)	99 (58.2)	
Mutated, n (%)	71 (41.7)	

*mCRC* metastatic colorectal cancer, *SD* standard deviation, *IDF* international diabetes federation, *BMI* body mass index, *KRAS* Kirsten rat sarcoma viral oncogene

The results revealed a statistically significant association between KRAS status according to primary cancer location and each of chol:HDL ratio and serum cholesterol (Table 2). For recto-sigmoid cancers, there was a statistically significant association between high chol:HDL ratio and KRAS mutation (OR 2.69, 95% CI 1.1–6.4, *p* = 0,02). In non recto-sigmoid cancers, high cholesterol was significantly associated with KRAS WT (OR 0.39, CI 0.15–0.97, *p* = 0.04), whilst there was a borderline association between MetS and KRAS mutation (OR 2.78, 95% CI 0.99–7.82, *p* = 0.05). There was no statistically significant association between BMI categories, hypertension, diabetes, statin use and KRAS status in any tumour location. From multivariable binary logistic regression chol:HDL ratio was the only significant predictor of KRAS status in recto-sigmoid cancers (OR 4.005, CI 1.223–13.116, *p* = 0.02). In non recto-sigmoid cancers triglycerides and HDL-chol were both significant predictors of KRAS status (OR 4.449, 95% CI 1.071–18.485, *p* = 0.04; OR 7.905, CI 1.121–55.744, p = 0.04, respectively) (Supplementary File Table 2). In multivariable analysis in colon cancers classified as right sided and left sided, we did not identify any statistically significant association between serum lipid profile and KRAS status. However, in right sided cancers MetS, defined with BMI ≥29 as cut-off of high waist circumference, was significantly associated with KRAS status (OR 6.000, 95% CI 1.501–23.991, *p* = 0.007) (Supplementary File Table 3).

**Table 2:** Clinical-pathological characteristics in 170 mCRC patients including lipid profile and metabolic parameters by tumour location and KRAS status – univariable analysis

	Total cancers	Total cancers	OR (95% CI), *p*-value^a^	Recto-sigmoid cancer	Recto-sigmoid cancer	OR (95% CI), *p*-value^a^	Non recto-sigmoid cancer	Non recto-sigmoid cancer	OR (95%CI), *p*-value^a^
	KRAS WT *n* = 99	KRAS mutated *n* = 71							
				KRAS WT *n* = 55	KRAS mutated *n* = 37		KRAS WT *n* = 44	KRAS mutated *n* = 34	
Gender male, n(%)	59 (59.59)	43 (60.5)	1.04 (0.55–1.94), 0.8	39 (70.9)	23 (62.7)	0.67 (0.27–1.63), 0.38	20 (45.4)	20 (58.82)	1.71 (0.69–4.23), 0.24
Age mean (SD)	65.6 (11.82)	67.2 (10.54)	0.5	63.6 (12.4)	66.3 (10.31)	0.3	68 (10.58)	68.2 (10.84)	0.94
Triglyceride ≥ 1.7 mmol/l, n (%)	36 (35.6)	30 (42.85)	1.28 (0.68–2.39), 0.4	24 (43.63)	15 (40.54)	0.88 (0.37–2.05), 0.8	12 (27.27%)	15 (45.45%)	2.10 (0.81–5.43), 0.12
Triglycerides mean (SD)	1.7 (1.42)	1.7 (0.81)	0.4	1.81(1.68)	1.67 (0.89)	0.9	1.48 (0.99)	1.62 (0.72)	0.2
Cholesterol ≥ 5 mmol/l, n (%)	61 (60.39)	37 (52.85)	0.67 (0.36–1.25), 0.21	34 (61.81%)	24 (64.9)	1.14 (0.47–2.71), 0.8	27 (61.4%)	13 (39.39)	**0.39 (0.15–0.97), 0.04**
Cholesterol mean(SD)	5.24 (0.95)	5.1 (0.90)	0.3	5.23 (1.00)	5.35 (0.88)	0.5	5.26 (0.89)	4.8 (0.87)	**0.03**
Chol:HDL ratio ≥ 3.5, n(%)	48 (48.5)	38 (54.28)	1.22 (0.66–2.25), 0.5	24 (43.6)	25 (67.6)	**2.69 (1.1–6.4), 0.02**	24 (54.5)	13 (38.2)	0.51 (0.20–1.28), 0.15
Chol:HDL ratio mean (SD)	3.7 (1.37)	3.9 (1.58)	0.4	3.64 (1.56)	4.07 (1.37)	**0.03**	3.73 (1.10)	3.62 (1.78)	0.18
LDL-chol ≥ 1.8 mmol/l, n (%)	91 (91.9)	64 (90.14)	0.80 (0.27–2.32), 0.6	48 (87.27)	35 (94.59)	2.55 (0.50–13.03), 0.24	43 (97.72)	29 (85.29)	0.13 (0.01–1.21), 0.08 ^b^
LDL-chol mean(SD)	2.83 (0.85)	2.97 (1.34)	0.8	2.71 (0.89)	3.09 (0.91)	0.06	2.97 (0.78)	2.85 (1.69)	0.06
HDL ≤ 1.03 mmol/dl in males and 1.29 mmol/dl in females, n(%)	16 (16.16)	11 (15.71)	1.05 (0.45–2.42), 0.90	7 (12.72)	8 (21.6)	0.52 (0.17–1.61), 0.2	9 (20.45%)	3 (8.82)	2.65 (0.66–10.70), 0.15
HDL-chol mean(SD)	1.55 (0.43)	1.45 (0.44)	0.08	1.57 (0.45)	1.43 (0.45)	0.11	1.53 (0.41)	1.47 (0.44)	0.42
Statin use	38 (38.4)	33 (46.5)	1.355 (0.733–2.506), 0.3	21 (38.2)	17 (45.9)	1.183 (0.511–2.739), 0.7	17 (38.6)	16 (47.1)	1.588 (0.642–3.929), 0.3
HbA1c ≥ 48 mmol/mol, n (%)^c^	16/71 (16.2)	14/40 (19.7)	1.85 (0.78–4.35), 0.1	19/38 (23.68)	9/22 (40.90)	0.1	7/33 (21.21)	5/18 (27.77)	0.7 ^b^
HbA1c mean (SD)^b^	41.97 (11.57)	43.33 (18.52)	0.8	42.54 (10.43)	39.80 (16.67)	0.8	41.32 (12.86)	47.71 (20.23)	0.45
Diabetes, n(%)	19 (19.2)	19 (27.14)	1.53 (0.74–3.17), 0.2	11 (20)	8 (21.62)	1.10 (0.39–3.07), 0.8	8 (18.18)	11 (33.33)	2.15 (0.75–6.15), 0.14
Hypertension, n(%)	61 (61.6)	49 (69.01)	1.38 (0.72–2.64), 0.3	33 (60)	23 (62.16)	1.09 (0.46–2.57),0.8	28 (63.6)	26 (78.78)	1.85 (0.68–5.06), 0.2
Metabolic Syndrome (IDF), n(%)	21 (21.21)	19 (26.76)	1.35 (0.66–2.76), 0.40	14 (25.45)	9 (24.32)	0.941 (0.358–2.472), 0.90	7 (15.90)	10 (29.41)	2.20 (0.738–6.577), 0.15
Metabolic Syndrome (waist circumference ≥ 29), n(%)	22 (22.22)	22 (30.98)	1.571 (0.787–3.136), 0.19	14 (25.45)	9 (24.32)	0.94 (0.358–2.472), 0.90	8 (18.18)	13 (38.23)	2.78 (0.99–7.82), 0.05
Normal (BMI ≥ 18.5 ≤ 24.9) n(%)	27/96^d^ (28.12)	16/69^e^ (23.18)		11 (20)	8/36^f^ (22.22)		16/41^g^ (39.02)	8/33^h^ (24.24)	
Overweight (BMI ≥ 24.9 ≤ 29.9) n(%)	41/96^d^ (42.70)	31/69^e^ (44.92)	0.77	26 (47.27)	16/36^f^ (44.44)	0.95	15/41^g^ (36.58)	15/33^h^ (45.45)	0.4
Obese (BMI ≥ 30), n(%)	28/96^d^ (29.16)	22/69^e^ (31.88)		18 (32.72)	12/36^f^ (33.33)		10/41^g^ (24.39)	10/33^h^ (30.30)	

*OR* odds ratio, *CI* confidence interval, *SD* standard deviation, *Chol:HDL* cholesterol:high density lipoprotein, *HbA1c* haemoglobin A1c, *IDF* international diabetes federation, *BMI* body mass index. Bold values indicate statistical significance

^a^P values for categorical and continuous characteristics were calculated by Chi-square and Mann-Whitney U tests, respectively

^b^Fisher’s exact test

^c^59 missing values

^d^3 values missing (underweight)

^e^2 values missing (underweight)

^f^one value missing (underweight)

^g^3 values missing (underweight)

^h^one value missing (underweight)

Associations between serum lipid profile and KRAS status were verified in a larger cohort of 201 patients with total cholesterol, chol:HDL ratio and HDL-cholesterol available. Results confirmed a significant association between high chol:HDL ratio and KRAS mutation in recto-sigmoid cancers (OR 3, CI 1.3–6.8, *p* = 0.009). In non recto-sigmoid cancers high cholesterol was significantly associated with KRAS WT (OR 0.3, CI 0.14–0.84, *p* = 0.01).

Among 201 patients KRAS mutations in codon 12 were the most frequently observed (31.3%). Codon 12 exhibited 5 mutational types and the most frequently observed was G12A both in recto-sigmoid and non recto-sigmoid cancers (31.1% and 31.6% respectively) (Table 3). We did not observe significant association between KRAS genotype and lipid profile or tumour location.

**Table 3:** KRAS codon, nucleotide change and amino acid change in 201 mCRC according to tumour location as recto-sigmoid cancer versus non recto-sigmoid cancer

KRAS codon	Nucleotide change	Amino acid change	n (%) among 201 mCRC cases	n (%) among 106 recto-sigmoid cancer cases	n (%) among 95 non recto-sigmoid cancer cases
12	c.34G > A	G12S	3 (1.5)	1 (1)	2 (2)
12	c.35G > T	G12V	17 (8.5)	8 (7.5)	9 (9.5)
12	c.35G > A	G12D	6 (3)	3 (2.8)	3 (3)
12	c.34G > T	G12C	8 (4)	4 (3.7)	4 (4)
	c.38G > A				
12	c.35G > C	G12A	29 (14)	17 (16)	12 (12.6)
	c.35G > A				
13	c.38G > A	G13D	2 (1)	–	2 (2)
13	c.38G > A	G13A	9 (4.5)	5 (4.7)	4 (4)
	c.37G > T				
61	c.181C > A	Q61K	3 (1.5)	–	3 (3)
	c.183A > C	Q61H			
146	c.436G > A	p.A146T	4 (2)	4 (3.7)	–
	c.436G > C	p.A146P			
	c.437C > T	p.A146P			

mCRC: metastatic colorectal cancer

**Fig. 1:**
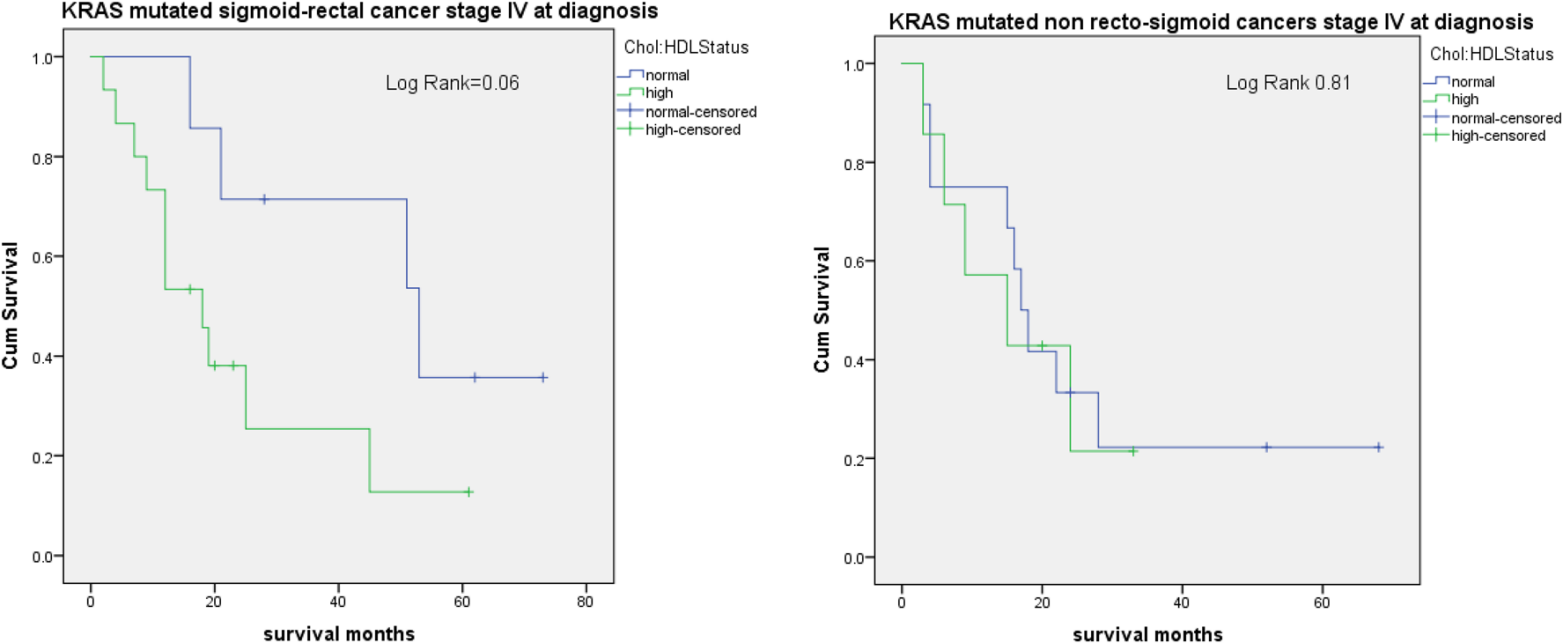
Survival analysis. Kaplan Meier survival curves of 22 patients with KRAS mutated recto-sigmoid cancer stage IV at diagnosis stratified by chol:HDL ratio (**a**); survival curves of 19 patients with KRAS mutated non recto-sigmoid cancers stage IV at diagnosis stratified by chol:HDL ratio (**b**). Patients with KRAS mutated recto-sigmoid cancer with normal chol:HDL ratio exhibited a better survival compared to patients with high chol:HDL ratio, not observed in non recto-sigmoid cancer

**Fig. 2:**
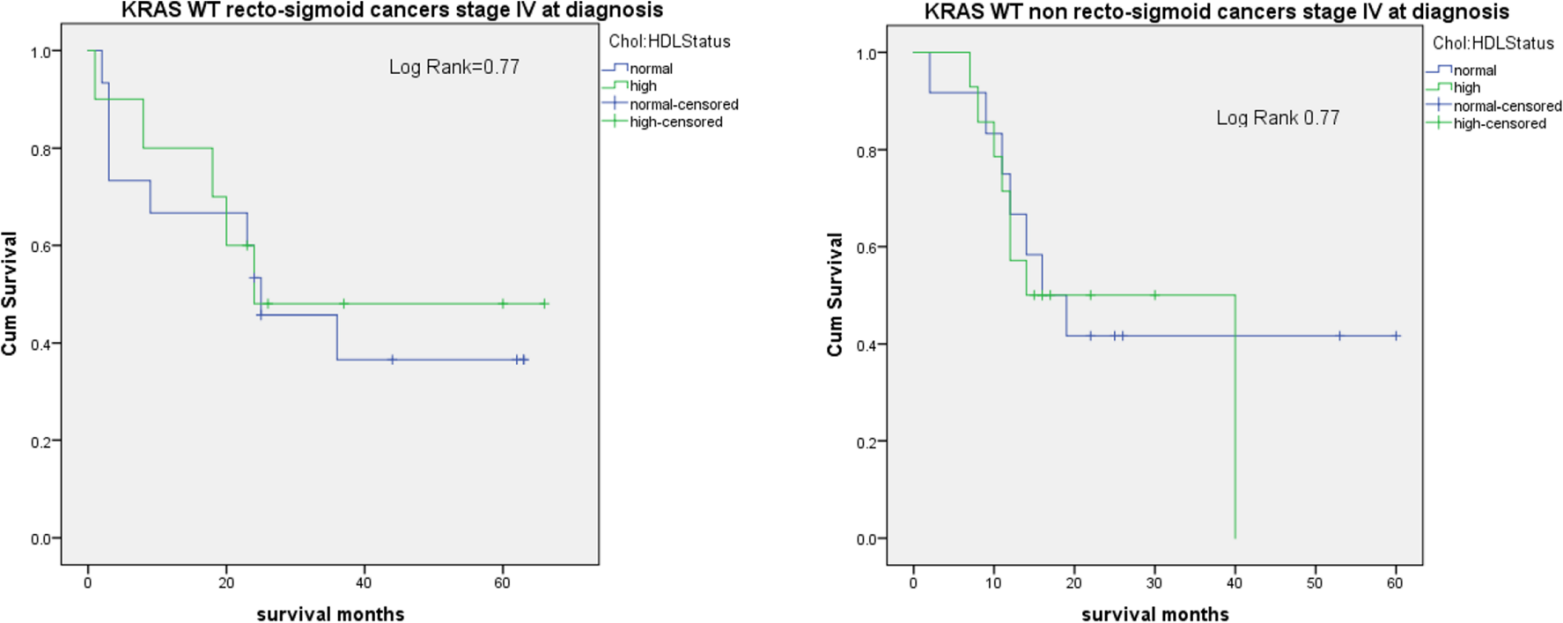
Survival analysis. Kaplan Meier survival curves of 26 patients with KRAS WT recto-sigmoid cancer stage IV at diagnosis stratified according to chol:HDL (**a**); Kaplan Meier survival curves of 26 patients with KRAS WT non recto-sigmoid cancer stage IV at diagnosis stratified according to chol:HDL (**b**). Results revealed no difference in survival in patients with KRAS WT cancer stratified by chol:HDL ratio for both tumour locations

Survival analysis was performed in 22 patients with KRAS mutated recto-sigmoid cancer stage IV at diagnosis. Results revealed a trend towards better survival in patients with low chol:HDL ratio compared to patients with high chol:HDL ratio (*p* = 0.06) (Fig. 1). No difference in survival was observed in patients with KRAS WT CRC for each tumour location (Fig. 2).

**Fig. 3:**
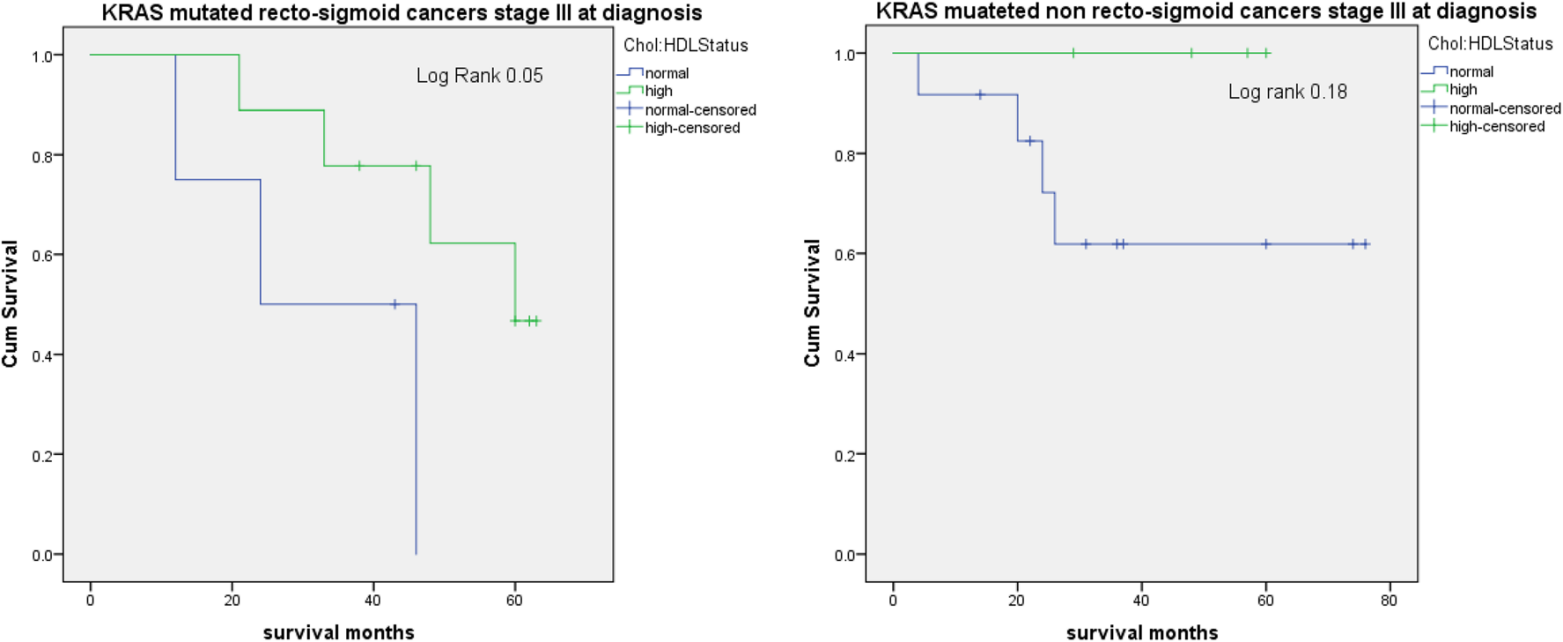
Survival analysis. Kaplan Meier survival curves in 13 patients with KRAS mutated recto-sigmoid cancer stage III at diagnosis stratified by chol:HDL ratio (**a**); Kaplan Meier survival curves in 16 patients with KRAS mutated non recto-sigmoid cancer stage III at diagnosis (**b**). Results show better survival in KRAS mutated recto-sigmoid cancer patients with high chol:HDL ratio compared to normal chol:HDL ratio

In 13 patients with KRAS mutated recto-sigmoid cancer stage III at diagnosis, we observed better survival in patients with high chol:HDL ratio (*p* = 0.05) (Fig. 3). We did not observe differences in 16 patients with KRAS mutated non recto-sigmoid cancer stage III at diagnosis according to chol:HDL ratio.

Out of 85 CRC patients stage III at diagnosis, 34 (40%) patients were on statin treatment. Out of 72 CRC patients stage IV at diagnosis, 31 (43.1%) patients were on statin treatment. We performed survival analysis in patients with stage III and stage IV CRC at diagnosis according to tumour location and statin treatment. No statistical difference in survival was observed.

## Discussion

Previous studies evaluating association between obesity/MetS and KRAS status have yielded inconclusive results [14–18]. In this study we investigated associations between serum lipid profile, BMI and metabolic factors with KRAS status according to primary tumour location. The present study differs from previous studies in the classification of primary tumour location, traditionally classified as right sided, left sided and rectal cancer. We employed a new classification of recto-sigmoid versus non recto-sigmoid cancers. Results of this exploratory study revealed association between lipid profile and KRAS status according to primary tumour location. In both univariable and multivariable analysis, there was a statistically significant association between chol:HDL ratio and KRAS mutation in recto-sigmoid cancers (*p* = 0.02). KRAS codon 13 mutations have been found to be significantly associated with high plasma cholesterol [24]. In this study we did not identify any associations between serum lipid profile and one specific KRAS mutation, likely related to the small sample size. Results of the present study suggest that dysregulated serum lipid profile may be implicated in KRAS mutated signaling pathways in a subset of KRAS mutated metastatic recto-sigmoid cancers, thus leading to the deduction that lipid lowering treatment may be effective in such subset of mCRC patients.

Clinical studies on the effectiveness of statin use after CRC diagnosis have yielded inconclusive results [25–28].A recent systematic review and meta-analysis evaluating statin use and mortality in CRC patients has highlighted that statin use pre and post CRC diagnosis improved overall and cancer specific survival in CRC patients. However, no statistical difference in all cause mortality in post-CRC diagnosis statin users in subgroup analysis by KRAS mutation status was identified, although there was a trend towards a reduction in all cause mortality in KRAS mutated CRC patients in treatment with statins after CRC diagnosis [29]. Therefore, there is an urgent clinical need to identify an appropriate subgroup of CRC patients that would benefit from statin treatment in association with adjuvant chemotherapy.

Retrospective cohort studies have shown that statin use in rectal cancer patients undergoing neoadjuvant chemoradiotherapy appears to confer higher pathological regression rates [30, 31]. Results of the present study have identified a subgroup of patients with KRAS mutated mCRC located at recto-sigmoid colon as a candidate subgroup of patients that may benefit from statin treatment in association with adjuvant chemotherapy.

Studies evaluating the role of dyslipidemia in CRC patients outcomes have yielded conflicting results [32, 33], with some studies highlighting better survival with high serum cholesterol.

In the present study survival analysis in 22 patients with KRAS mutated recto-sigmoid cancer stage IV at diagnosis revealed a trend towards better survival in patients with low chol:HDL ratio compared to patients with high chol:HDL ratio (*p* = 0.06). In a subgroup analysis comparing mCRC patients in treatment with statins versus non statin users stratified by tumour location (recto-sigmoid versus non recto-sigmoid colon, right colon versus left colon, colon versus rectum) and KRAS status, we did not observe improved survival in CRC patients in treatment with statins. It is noteworthy that mean cholesterol and chol:HDL ratio in patients with recto-sigmoid cancers treated with statins were above therapeutic target levels (5.1 mmol/l and 3.6 respectively), suggesting that suboptimal treatment of dyslipidemia may have been responsible of the lack of expected improved survival observed in such patients in treatment with statins (Supplementary File Table 4). It is plausible that the effect of statins on a subset of CRC patients outcomes is related to its lipid lowering effect.

In patients with KRAS mutated recto-sigmoid cancer stage III at diagnosis, high chol:HDL ratio was associated with better survival. These results are in line with previous studies demonstrating that dyslipidemia resulted in improved survival in patients with non-metastatic CRC [34]. The mechanisms underlying the protective effect of dyslipidemia in non-mCRC remain elusive. Serum cholesterol levels have been demonstrated to decline prior to diagnosis of CRC [35], likely related to actively proliferating and aggressive cancer cells, which utilize host lipids for proliferation and survival. High serum lipid levels, in the setting of non-mCRC, may reflect a less aggressive cancer phenotype.

In univariable analysis, high serum cholesterol level was associated with KRAS WT in non recto-sigmoid cancers (*p* = 0.04). Given the retrospective nature of this study waist circumference could not be measured, resulting in likely underestimation of the MetS group. Therefore, in order to increase patient group with MetS, we reduced BMI cut off to ≥29, resulting in 2 more patients with MetS for each tumour location. Employing a lower BMI cut off for obesity at ≥29 as assumption of high waist circumference, there was a borderline association between MetS and KRAS mutation in non recto-sigmoid cancers (*p* = 0.05), whilst no significant association was identified employing cut off ≥30 as per IDF guidelines.

The prognostic and predictive role of primary tumour location classified as right sided versus left sided colon cancers has recently been highlighted. In the present study, in multivariable analysis in colon cancers classified as right sided and left sided, we did not identify any statistically significant association between serum lipid profile and KRAS status. However, in right sided cancers MetS, defined with BMI ≥29 as cut-off of high waist circumference, was significantly associated with KRAS status (OR 6.000, 95% CI 1.501–23.991, *p* = 0.007).

In-vitro studies have suggested that statins may interfere with KRAS downstream signaling pathways via inhibition of KRAS prenylation essential for membrane translocation of KRAS and activation of signaling pathways. Experimental data has highlighted that statins can reverse cetuximab resistance in KRAS mutant CRC [36] and enhance chemosensitivity to 5-fluorouracil inducing epigenetic reprogramming [37]. In-vitro studies have demonstrated that targeting cholesterol synthesis results in sensitizing resistant cancer cells to EGFR inhibitors [38, 39]. However, one randomized clinical trial has failed to demonstrate the efficacy of statins in KRAS mutated mCRC patients in treatment with Cetuximab [40]. Results of this study raise the hypothesis of a possible enhancing chemosensitivity effect of statins in a subset of KRAS mutated mCRC patients with recto-sigmoid cancer.

Lipids are an essential component of plasma membrane involved in cancer cell signaling and cancer metabolism and their role in KRAS-mediated cancers is emerging [41, 42]. KRAS proteins and signaling pathways have received broad attention for anticancer therapy. Several therapeutic approaches targeting oncogenic RAS have been explored, including inhibition of enzymes involved in post-translational modification of RAS proteins [43], siRNA for silencing KRAS isoforms [44] amongst others. Nevertheless, to date there are no effective anti KRAS therapies.

**Fig. 4:**
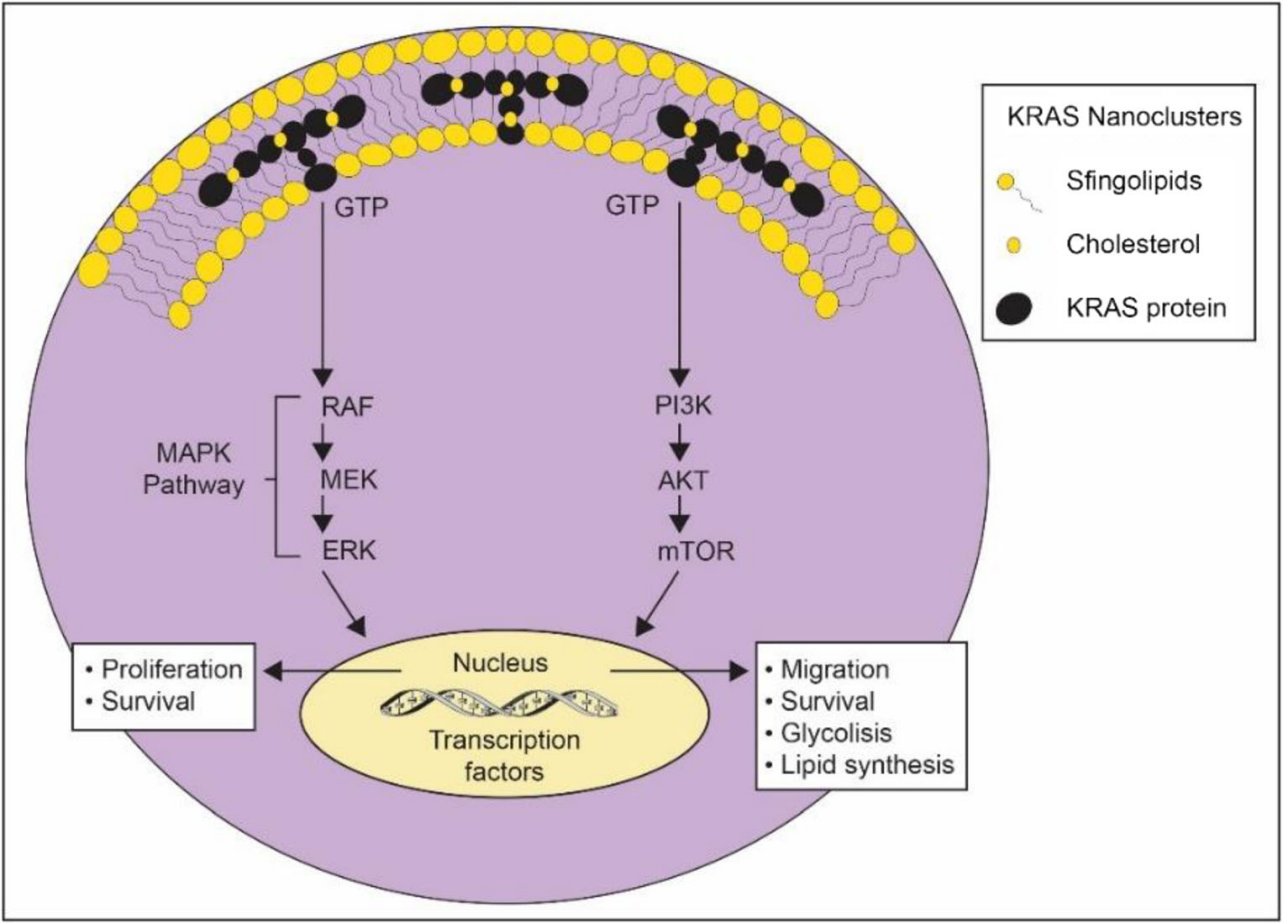
Representation of KRAS nanoclusters in plasma membrane and signaling pathways. GTP: guanosin 5′-triphosphate; MAPK: Mitogen-activated protein kinase; RAF: Rapidly Accelerated Fibrosarcoma; MEK: mitogen-activated protein kinase kinase; ERK: Extracellular signal-Regulated Kinase; PI3K: Phosphoinositide 3-kinase; AKT: serine/threonine protein kinase; mTOR: mammalian target of rapamycin

KRAS proteins assemble on the plasma membrane in nanoclusters [45–47], essential for effector binding and activation of MAPK pathway [48] and phosphatidyl inositol 3-kinase (PI3K)/AKT/mTOR signaling pathway [49], resulting in cell proliferation and survival. Cancers exhibit a reprogrammed metabolic phenotype to supply high energetic demands and biosynthetic pathways, consisting of increased avidity for glucose, activation of aerobic glycolysis, reprogramming of amino-acid metabolism and altered lipid metabolism and fatty acid oxidation [50, 51]. Mutated KRAS has recently emerged as a key driver gene of metabolic reprogramming [41, 42, 52–54]. Therapies targeting metabolic reprogramming have recently been suggested with variable outcomes depending on local environment, highlighting that the microenvironment plays a key role in KRAS mutant metabolic remodelling [55]. Figure 4 depicts KRAS distribution in the plasma membrane and downstream effects.

Mutant KRAS driven cancers have been implicated in cell signaling and tumour metabolic remodelling in an environment dependent manner, mediated through cross-talk with surrounding stromal compartment [56] and differing according to tissue of origin [57]. According to the results of the present study, we could postulate that dysregulation of multiple systemic metabolic factors is implicated in KRAS-driven metabolic reprogramming in non recto-sigmoid cancers, whilst in cancers with a distal location serum dyslipidemia may facilitate KRAS related cancer progression. The difference between the two subsites may reflect diversities in tumour microenvironment (TME), with a TME in distal colon favoring KRAS related tumor progression in patients with serum dyslipidemia. In vitro studies employing three-dimensional (3D) cancer organoid models derived from recto-sigmoid cancers and non recto-sigmoid cancers are warranted to understand the role of lipids in KRAS mutated signaling pathways and cancer metabolism within the 2 different tumour locations. These in vitro organ-like cultures reproduce intestinal tissue microenvironment proteins and lipids [58], allowing to understand interplay between tumour cells and microenvironment.

CRC is a heterogeneous disease and it is plausible that the effect of statins may differ according to tumour location and molecular subtype. Retrospective studies and prospective clinical trials evaluating the effect of statins on patients stratified by tumour location and KRAS status are lacking. Therefore, a new approach in the design of clinical trials is urgently needed taking into consideration primary tumour location. According to the results of this study, we suggest clustering together rectal and sigmoid cancers versus non recto-sigmoid cancers stratified by KRAS status.

Clinical implications of the results of this study are multiple. Firstly, it lays the foundations to improve colon cancer treatment through optimal lipid lowering treatment in association with adjuvant chemotherapy in a subgroup of patients with KRAS mutated metastatic recto-sigmoid cancer.

Secondly, it offers insights on possible prognostic and predictive biomarkers in mCRC. Chol:HDL ratio could represent a valuable adjunctive metabolic prognostic and predictive biomarker in a subset of KRAS mutated mCRCs located at sigmoid-rectum. In this study in non recto-sigmoid cancers high serum cholesterol was associated with KRAS WT. Evaluation of high serum cholesterol as a predictive biomarker of response to anti EGFR therapies in non recto-sigmoid cancers in retrospective/prospective studies may warrant further evaluation.

Finally, serum lipid profile could be a valuable risk stratification tool to help identify high risk individuals to be implemented in current CRC screening and surveillance strategies. This hypothesis needs to be explored in large multicentre studies.

A limitation of this study is the relatively small sample size. Precise estimates were not feasible, such as high odds ratio and wide confidence intervals that include the one (the null hypothesis value) observed when evaluating associations between MetS and KRAS status in non recto-sigmoid cancers.

In conclusion, chol:HDL ratio was significantly associated with KRAS mutation in metastatic recto-sigmoid cancers. Lipid lowering treatment may be effective in a subset of patients with KRAS mutated metastatic recto-sigmoid cancer. Results of this study need to be validated in a larger multicentre cohort. Randomized controlled trials employing our novel tumour location classification are warranted to investigate the effectiveness of statins in a subgroup of mCRC patients.

Results of this study could lead to improved individualized screening, surveillance and treatment strategies.

## Electronic supplementary material

ESM 1 (DOCX 16.1 kb)

ESM 2 (DOCX 18.2 kb)

ESM 3 (DOCX 23 kb)

ESM 4 (DOCX 13.9 kb)
