# Supplementary material for: KRAS Status is Associated with Metabolic Parameters in Metastatic Colorectal Cancer According to Primary Tumour Location
Source: Pathol Oncol Res. 2020 Jun 27;26(4):2537–48. doi: 10.1007/s12253-020-00850-y (PMC7471139; doi:10.1007/s12253-020-00850-y)
Supplement: Supplementary file 1 — (DOCX 16.1 kb) [file 12253_2020_850_MOESM1_ESM.docx]

Supplementary File Table 1

Multivariable analysis for KRAS status according to tumour location as recto-sigmoid *versus* non recto-sigmoid cancers.

| **Variables** | **Total cancers**  **n=170** |  | **Recto-sigmoid cancers**  **n= 92** |  | **Non recto-sigmoid cancers**  **n=78** |  |
| --- | --- | --- | --- | --- | --- | --- |
|  | **OR (95%CI)** | **p-value** | **OR (95%CI)** | **p-value** | **OR (95%CI)** | **p-value** |
| **Age (<60 years *vs.* > 60 years)** | 1.009(0.977-1.041) | 0.58 | 1.028 (0.982-1.077) | 0.23 | 0.989(0.935-1.046) | 0.70 |
| **Sex (male *vs.* female)** | 0.866(0.441-1.699) | 0.67 | 0.501 (0.180-1.395) | 0.18 | 1.159(0.354-3.797) | 0.80 |
| **Triglyceride (<1.7 mmol/l *vs.* ≥ 1.7 mmol/l)** | 1.024(0.475-2.207) | 0.95 | 0.484(0.144-1.624) | 0.24 | 4.449(1.071-18.485) | **0.04** |
| **Cholesterol (<5 mmol/l *vs.* ≥ 5 mmol/l)** | 0.679(0.341-1.354) | 0.27 | 0.803(0.281-2.291) | 0.68 | 0.438(0.135-1.418) | 0.16 |
| **Chol:HDL (< 3.5 *vs.* ≥3.5)** | 1.368(0.633-2.957) | 0.42 | 4.005(1.223-13.116) | **0.02** | 0.501(0.129-1.939) | 0.31 |
| **HDL-cholesterol (≤ 1.03 mmol/dl *vs.* > 1.03 mmol/dl in males; ≤1.29 mmol/dl *vs.* > 1.03 mmol/dl in females)** | 1.303(0.493-3.442) | 0.59 | 0.405(0.099-1.650) | 0.20 | 7.905(1.121-55.744) | **0.03** |
| **LDL (< 1.8 mmol/l *vs.* ≥ 1.8 mmol/l)** | 0.835(0.256-2.720) | 0.76 | 1.851(0.274-12.497) | 0.52 | 0.261(0.023-2.984) | 0.2 |
| **Statin use (treatment *vs.* no treatment)** | 1.079(0.513-2.270) | 0.84 | 1.605(0.501-5.144) | 0.42 | 0.865(0.262-2.852) | 0.81 |
| **Diabetes (present *vs.* absent)** | 1.203(0.524-2.764) | 0.66 | 0.864(0.242-3.087) | 0.82 | 1.268(0.287-5.607) | 0.7 |
| **Hypertension (present *vs.* absent)** | 1.148(0.544-2.422) | 0.71 | 1.090(0.385-3.089) | 0.87 | 1.417(0.357-5.630) | 0.6 |
| **BMI ( < 25 *vs.* ≥ 25)** | 0.857(0.409-1.797) | 0.68 | 0.396(0.127-1.139) | 0.11 | 0.714(0.189-2.702) | 0.6 |
| **Metabolic syndrome, BMI ≥29 as assumption for waist circumference (present *vs.* absent)** | 1.523(0.663-3.495) | 0.32 | 0.995(0.268-3.689) | 0.9 | 3.908(0.825-18.519) | 0.08 |

OR, odds ratio; CI, confidence interval; Chol:HDL, cholesterol: high-density lipoprotein; HDL, high-density lipoprotein; LDL; low-density lipoprotein; BMI, body mass index. Bold values indicate statistical significance.
