# Supplementary material for: KRAS Status is Associated with Metabolic Parameters in Metastatic Colorectal Cancer According to Primary Tumour Location
Source: Pathol Oncol Res. 2020 Jun 27;26(4):2537–48. doi: 10.1007/s12253-020-00850-y (PMC7471139; doi:10.1007/s12253-020-00850-y)
Supplement: Supplementary file 3 — (DOCX 23 kb) [file 12253_2020_850_MOESM3_ESM.docx]

Table 3

Multivariable analysis for KRAS status according to tumour location as right colon cancers and left colon cancers.

| **Variables** | **Right colon cancers**  **n=65** |  | **Left colon cancers**  **n= 48** |  |  |  |
| --- | --- | --- | --- | --- | --- | --- |
|  | **OR (95%CI)** | **p-value** | **OR (95%CI)** | **p-value** |  |  |
| **Age (<60 years *vs.* > 60 years)** | 0.960 (0.310-2.968) | 0.94 | 0.900 (0.225-3.604) | 1 |  |  |
| **Sex (male *vs.* female)** | 2.028 (0.747-5.509) | 0.163 | 2.813 (0.736-10.751) | 0.124 |  |  |
| **Triglyceride (<1.7 mmol/l *vs.* ≥ 1.7 mmol/l)** | 1.825 (0.644-5.169) | 0.25 | 2.908 (0.797-20.602) | 0.10 |  |  |
| **Cholesterol (<5 mmol/l *vs.* ≥ 5 mmol/l)** | 0.386 (0.141-1.054) | 0.06 | 0.982 (0.268-3.602) | 1 |  |  |
| **Chol:HDL (< 3.5 *vs.* ≥3.5)** | 0. 500 (0.186-1.347) | 0.16 | 1.500 (0.428-5.259) | 0.52 |  |  |
| **HDL-cholesterol (≤ 1.03 mmol/dl *vs.* > 1.03 mmol/dl in males; ≤1.29 mmol/dl *vs.* > 1.03 mmol/dl in females)** | 2.148 (0.488-9.453) | 0.47 | 0.786(0.167-3.707) | 1 |  |  |
| **LDL (< 1.8 mmol/l *vs.* ≥ 1.8 mmol/l)** | 0.452 (0.077-2.658) | 0.42 | 0.375 (0.047-2.969) | 0.56 |  |  |
| **Statin use (treatment *vs.* no treatment)** | 1.543 (0.580-4.104) | 0.38 | 2. 083 (0.565-7.676) | 0.31 |  |  |
| **Diabetes (present *vs.* absent)** | 2.045 (0.642-6.513) | 0.22 | 0.778(0.137-4.419) | 1 |  |  |
| **Hypertension (present *vs.* absent)** | 1.304 (0.438-3.885) | 0.63 | 1.974(0.515-7.558) | 0.31 |  |  |
| **BMI ( < 25 *vs.* ≥ 25)**  **Metabolic syndrome, BMI ≥30 as assumption for waist circumference (present *vs.* absent)** | 0.833 (0.665-5.054)  3.913 (0.951-16.109) | 0.23  0.048 | 1.528 (0.350-6.674)  2.400 (0.666- 8.643) | 0.72  0.25 |  |  |
| **Metabolic syndrome, BMI ≥29 as assumption for waist circumference (present *vs.* absent)** | 6.000 (1.501-23.991) | **0.007** | 2.091 (0.587-7.448) | 0.25 |  |  |

OR, odds ratio; CI, confidence interval; Chol:HDL, cholesterol: high-density lipoprotein; HDL, high-density lipoprotein; LDL;

low-density lipoprotein; BMI, body mass index. Bold values indicate statistical significance.
