# Supplementary material for: KRAS Status is Associated with Metabolic Parameters in Metastatic Colorectal Cancer According to Primary Tumour Location
Source: Pathol Oncol Res. 2020 Jun 27;26(4):2537–48. doi: 10.1007/s12253-020-00850-y (PMC7471139; doi:10.1007/s12253-020-00850-y)
Supplement: Supplementary file 4 — (DOCX 13.9 kb) [file 12253_2020_850_MOESM4_ESM.docx]

Supplementary File Table 4.

Cholesterol and chol:HDL serum level in patients with recto-sigmoid cancers

|  | Statin treatment | No statin treatment |
| --- | --- | --- |
| Cholesterol, mean (SD) | 5.1 (1.03) | 5.3 (0.88) |
| Chol:HDL, mean (SD) | 3.6 (1.09) | 3.9 (1.74) |
